# Ignatzschineria larvae Bacteremia in a Patient With Chronic Leg Ulcer: A Case Report and Review of the Literature

**DOI:** 10.7759/cureus.36612

**Published:** 2023-03-23

**Authors:** Saudina Demurtas, Emmanuela Pareti, Matiar Madanchi

**Affiliations:** 1 Department of Internal Medicine, Ente Ospedaliero Cantonale, Locarno, CHE; 2 Department of Emergency, Ente Ospedaliero Cantonale, Locarno, CHE; 3 Department of Dermatology, University Hospital of Basel, Basel, CHE

**Keywords:** bacteremia, socio-economic factors, combination antibiotic therapy, chronic leg ulcers, ignatzschineria larvae

## Abstract

*Ignatzschineria larvae* (*I. larvae*) is a bacterium found in the digestive tract of some flies. A few cases of bacteremia by *I. larvae* are described in the literature. We present the case of a patient with chronic leg ulcer and poor hygienic and social conditions, who presented with bacteremia from *I. larvae*. As there are few cases described in the literature, there are no guidelines yet for the treatment of this bacteremia. We report a short review of the literature below.

## Introduction

*Ignatzschineria larvae* (*I. larvae*) is a bacterium found in the digestive tract of some flies (for example Wohlfahrtia magnifica) that infects animal wounds, first isolated in the early 2000s [1-3].

*I. larvae* bacteremia is associated with a parasitosis that is usually uncommon in humans, but more common in grazing animals [1].

Fewer than 10 cases of bacteremia from *I. larvae* are reported in the literature. In contrast, numerous infections/bacteremia from other *Ignatzschineria *species (*I. indica*, *ureiclastica*) have been described.

The case we report would be the second case of bacteremia from *I. larvae* in a human in Switzerland and the seventh worldwide.

## Case presentation

We present a case of an 81-year-old patient residing in Switzerland, known to have bilateral lower extremity ulcers who presented to the emergency department for clinical worsening and decline in general condition. The patient is also known to have socioeconomic problems (until a week earlier, he was living at a home for the elderly from which he had self-dismissed due to economic issues, returning to his home in poor condition).

At the hospital, the patient presented as hemodynamically stable and apyretic, with blood pressure values in the normal range. On admission, hematochemical examinations showed moderate C-reactive protein (CRP) elevation (polymerase chain reaction (PCR) 54mg/L; normal range <10 mg/L) with neutrophilic leukocytosis (10.28 x10E9/L; normal range <10.00) and blood culture were performed.

**Figure 1:**
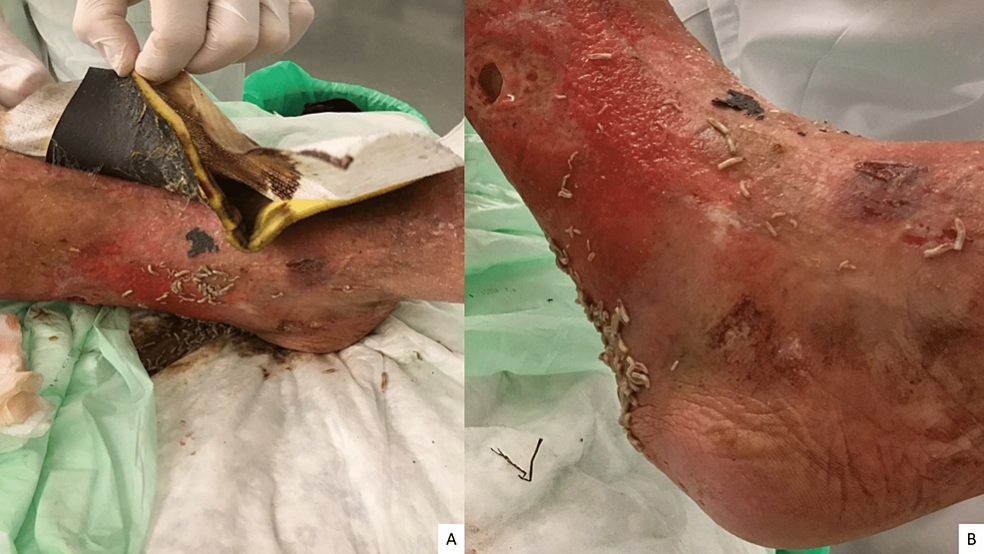
The presence of larvae at the level of the right leg on the arrival of the patient in the emergency department (A); In addition to the presence of larvae, an ulcer can be seen (B).

The physical exam showed poor hygienic conditions, infected and necrotic ulcers on the lower limbs, and malodorous ulcers with bone exposure at the left medial supra-malleolar level with the presence of larvae (see Figure 1). The wounds were debrided, and all the visible larvae were removed.

After surgical debridement, intravenous treatment with Ertapenem 1g/day was set up. The choice of antibiotic was influenced by the patient’s previous antibiogram resistance. The blood culture became positive for the gram-negative bacterium on the second day of hospitalization. The molecular investigation reveal that the bacteria was *I. larvae* (our antibiogram showed sensitivity for Ertapenem, Ceftriaxone, Levofloxacin, and Trimethoprim/Sulfamethoxazole). After infectious diseases consultation and in the absence of guidelines for the treatment of *I. larvae*, we continued the treatment with Ertapenem for a total of two weeks. After two weeks of treatment, we noticed a clear clinical and laboratory improvement. For this reason, we modified the antibiotic therapy by setting up an oral treatment with sulfamethoxazole/trimethoprim 800/160 2x/day for a total of three months.

During the hospitalization, an angiological evaluation was performed. That showed an obstructive arterial disease with probable right femoral occlusion and left femoral stenosis with little reduced distal perfusion, for that reason revascularization was not required.

**Figure 2:**
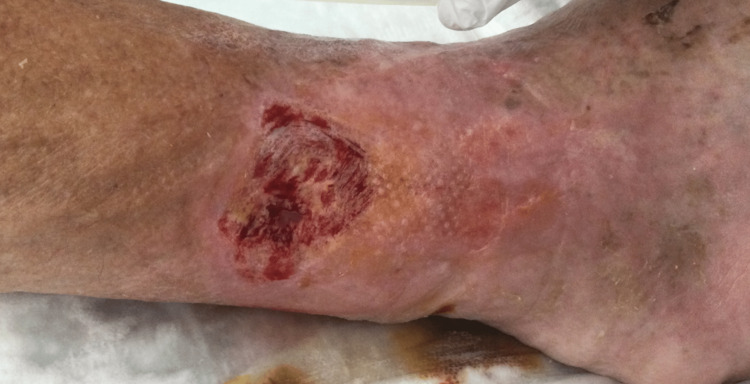
Ulcer on the right leg after three weeks of hospitalization.

After about three total weeks of hospitalization, the ulcers were much improved (see Figure 2). We discussed with the patient and his family about his discharge and in agreement with him and his family we believe that returning to the elderly home is the safest and most appropriate solution, also to allow to continue adequate dressing of the ulcers.

## Discussion

Cutaneous myiasis is a parasitosis of the skin caused by the larvae of certain species of flies. There are three types of myiasis: furuncular, wound, and migratory [2]. Human myiasis is most common in poor countries in tropical or sub-tropical regions. It could also be found in patients who have travelled to these areas [2].

Open wound myiasis, on the other hand, occurs in patients who are homeless or live in poor social conditions. Such conditions put them at risk of being infested with fly larvae [2].

There are three genera of Ignatzschineria: *larvae*, *indica*, and *ureiclastica*. All three genera are non-sporulating, non-haemolytic, non-motile, rod-shaped bacteria that were first isolated in 2001 from larvae of Wohlfahrtia magnifica, a parasitic flesh fly causing myiasis in several animal species and occasionally in humans [4]. Originally the name of this bacterium was simply *Schineria *in honour of Ignatz Rudolph Schiner (1813-1873), who first described the fly Wohlfahrtia magnifica in 1862 [3]. Subsequently, Toth et al. proposed the change of the name to *Ignatzschineria *because the first name was previously used in 1857 for another species (*Schineria Rondani*) [3].

Bacteremia by *I. larvae* is little known, and fewer than 10 cases have been described worldwide. Partly because this bacterium was first described only 20 years ago, and its identification is difficult with standard methods.

In the literature, there are six cases of *I. larvae*’s bacteremia (see Table 1). Our case is the seventh. In all seven cases, we are dealing with male patients (100% M), from 18 to 90 years old (mean age was 61 years old). Of all the seven cases, five patients had chronic ulcers (71.4%), and five patients had a psychiatric disorder (drug addiction, alcoholism, schizophrenia) (71.4%). In all cases, blood cultures showed positivity for *I. larvae*. All cases were treated with different antibiotics and for different durations. All patients had maggots in the ulcers. At admission, four patients received combined antibiotic therapy (57.1%), and the other three received one antibiotic (42.9%).

**Table 1:** A review of the literature.

References	Maniam et al. [4]	Nadrah et al. [5]	Reed et al. [6]	Grasland et al. [7]	DiFranza et al. [8]	Berthod et al. [9]	Our case
Cases, n	1	1	1	1	1	1	1
Age of patients, years	68	18	66	50	58	88	80
Gender	Male	Male	Male	Male	Male	Male	Male
Origin	American	Asian	Afroamerican	Unknown	American	Swiss	Swiss
Chronic ulcers	Yes	No	Yes	Yes	Yes	No	Yes
Poor socioeconomic conditions	Yes	Yes	Yes	Yes	Yes	Yes	Yes
Co-morbidities	Hypertension; Heroin abuse	-	Schizophrenia Cocaine and opioids abuse	Alcoholism abuse	Hypertension; Bipolar disorder; Alcoholism and polysubstance abuse	Diabetes Type II	Behaviour disturb; Chronic arteriopathy
Positive blood culture	Yes	Yes	Yes	Yes	Yes	Yes	Yes
Treatment	Cefepime + vancomycin i.v, and then levofloxacin per os	Flucloxacillin + ciprofloxacin, then tigecycline + vancomycin and then imipenem + vancomycin	Tazobactam and then meropenem	Ceftriaxone + gentamicin, then ceftriaxone and at the discharge Amoxicillin/clavulanic acid	Tazobactam + vancomycin and then levofloxacin + doxycycline	Amoxicillin/clavulanic acid, then tazobactam and then Trimethoprim/sulfamethoxazole	Ertapenem i.v and then Trimethoprim/sulfamethoxazole per os

## Conclusions

In our case, we were confronted with an 81-year-old male patient in the poor hygienic condition who had infected ulcers on his lower limbs. The presence of chronic ulcers, combined with precarious living conditions, seems to favour the infestation.

It is therefore essential in patients who present with wounds contaminated by larvae to systematically perform blood cultures, signalling the laboratory to search for the larvae as a possible pathogenic agent. Consequently, the possible *I. larvae* bacteraemia must be kept in mind when choosing the antibiotic. There is a possibility that the number of bacteremias is higher than that documented because only in the last few years have the diagnostic investigations improved.

It is, therefore, necessary to create guidelines for a systematic approach to *I. larvae*’s bacteremia, in order to avoid inadequate treatment, thus increasing healing time and the risk of creating more antibiotic resistance it is also necessary to schedule a close follow-up to monitor the progress of the wounds, especially in patient at risk (alcohol and drug abuse, mental illness, poor social conditions).
